# A novel RNA-based *in situ* hybridization to detect Seneca Valley virus in neonatal piglets and sows affected with vesicular disease

**DOI:** 10.1371/journal.pone.0173190

**Published:** 2017-04-10

**Authors:** Talita P. Resende, Douglas G. Marthaler, Fabio A. Vannucci

**Affiliations:** 1 Department of Veterinary and Biomedical Sciences, College of Veterinary Medicine, University of Minnesota, Saint Paul, Minnesota, United States of America; 2 Veterinary Diagnostic Laboratory, College of Veterinary Medicine, University of Minnesota, Saint Paul, Minnesota, United States of America; University of Maryland at College Park, UNITED STATES

## Abstract

Seneca Valley virus (SVV) is the causative agent of an emerging vesicular disease in swine, which is clinically indistinguishable from other vesicular diseases such as foot-and-mouth disease. In addition, SVV has been associated with neonatal mortality in piglets. While a commercial SVV qRT-PCR is available, commercial antibodies are lacking to diagnose SVV infections by immunohistochemistry (IHC). Thus, a novel *in situ* hybridization technique—RNAscope (ISH) was developed to detect SVVRNA in infected tissues. From a total of 78 samples evaluated, 30 were positive by qRT-PCR and ISH-RNA, including vesicular lesions of affected sows, ulcerative lesions in the tongue of piglets and various other tissues with no evidence of histological lesions. Nineteen samples were negative for SVV by qRT-PCR and ISH-RNA. The Ct values of the qRT-PCR from ISH-RNA positive tissues varied from 12.0 to 32.6 (5.12 x 10^6^ to 5.31 RNA copies/g, respectively). The ISH-RNA technique is an important tool in diagnosing and investigating the pathogenesis of SVV and other emerging pathogens.

## Introduction

Seneca Valley virus, which belongs to the Senecavirus A species, has been isolated from pigs since 1988 and was reported as picornavirus-like particle [1] until 2002, when it was fully characterized as an oncolytic picornavirus [2]. Historically, SVV infections have been associated with swine idiopathic vesicular disease (SIVD) in Canada and United States [1,2]. Recently, the virus has been associated with vesicular disease and neonatal mortality in commercial swine herds in Brazil [3–5], United States [6,7] and China [8]. In sows, vesicular lesions and coalescing erosions are found on the coronary bands and snouts. Vesicular lesions in affected pigs are indistinguishable from important animal diseases with devastating economic impact in the food animal industry such as swine vesicular disease (SVD), vesicular stomatitis (VS), vesicular exanthema of swine (VES) and foot-and-mouth disease (FMD). SVV infections also have been associated with acute neonatal mortality, recently named epidemic transient neonatal losses (ETNL)[3,6,8,9]. The epidemiology of SVV is not well understood, but SVV nucleic acid has been identified in environmental samples, feces and intestines of mice, and in house flies collected from affected herds [7]. Therefore, rodents and flies may represent potential vectors involved in SVV transmission, and the virus is likely persistent in the environment [7].

During recent outbreaks of vesicular disease in Brazil and USA in 2015, SVV was assumed to be the causative agent of SIVD and ETNL based on virus detection by RT-PCR associated with clinical signs of vesicular lesions [4,6,9]. However, it is well known that PCR detection of a pathogen in a tissue does not mean that the agent caused the lesions or disease. To establish that a pathogen is the causative agent of disease, it is necessary to identify the presence of the pathogen and its association with morphological changes within the tissues [10]. Immunohistochemistry (IHC) and *in situ* hybridization (ISH) are the main methodologies used for this purpose. While IHC identifies a specific protein associated with the pathogen, ISH detects the pathogen’s nucleic acid within the tissue sections. Immunohistochemistry is limited on availability of species-specific antibodies and performance variation among suppliers and batches [10], and a commercial antibody for SVV is lacking.

*In situ* hybridization recognizes specific nucleic acid sequence of the pathogen by the use of a probe, which is a reverse complementary of the pathogen sequence. Similar to PCR, ISH has the advantage of identifying nucleic acids within the lesions. Historically, the challenge of classical *in situ* hybridization is its lack of sensitivity, especially involving the lower labelling efficiency of different haptens (i.e. dioxigenin and dinitrophenol) to short oligonucleotide sequences [10]. Recently, a novel ISH-RNA technology (RNAscope®, Advanced Cell Diagnostics Inc.) has been developed, which describes the visualization of a single-molecule through the use of hybridization coupled with a signal amplification system to increase the sensitivity of the technique [11]. The ISH-RNA technique has been valuable as a rapid diagnostic response to SVV outbreaks in the US and Brazil in 2015, since the specific commercial SVV antibodies were lacking, to the best of the authors’ knowledge. The objectives of this study were to evaluate the detection of SVV using ISH-RNA technique in tissue samples compared with the qRT-PCR results obtained from infected pigs and determine the replication site of SVV within infected tissues.

## Materials and methods

### Tissue selection

Tissues are routinely submitted to the Veterinary Diagnostic Laboratory, University of Minnesota (MNVDL) for diagnostic investigations. Upon arrival, nucleic acid was extracted from fresh tissues and tested for a variety of pathogens by PCR while the formalin-fixed samples were embedded in paraffin for histological evaluation. The inclusion criteria for testing samples with the ISH-RNA technique was based on the qRT-PCR results from retrospective outbreaks of SVV in sows and neonatal piglets. Samples with Ct values lower than 36 were considered qRT-PCR positive for SVV and were, therefore, selected to be analyzed by the in situ technique. The availability and diversity of tissues were dependent on the submitter. As a result, tissue selection varied by sample date, which included the number and type of tissues submitted.

Non-affected pigs from both affected and non-affected herds were used as negative controls and showed negative SVV results by qRT-PCR (S1 Table). A total of 78 tissues were tested, including 59 samples from 18 affected pigs and 19 samples from 15 non-affected pigs. Affected sows were exhibiting clinical signs consistent with vesicular disease in the snouts and coronary band, while affected litters of piglets showed transient sudden death suggestive of ETNL.

### Quantitative RT-PCR (qRT-PCR)

Fresh tissue samples were tested with a commercially available qRT-PCR assay according to manufacturer’s instructions (Tetracore Inc, Gaithersburg, Md.). In order to determine the limit of detection for qRT-PCR assay, a SVV cell culture isolate (D15-046416-2 P2) was tittered (5.62 x 10^7^ TCID_50_), and 10 fold dilutions (10^−1^ to 10^−10^) were made, and RNA was extracted, using previously described methods [12] of the Tetracore EZ-SVV qRT-PCR. The dilutions were tested on multiple days by different individuals to determine reproducibility and the detection limit, which indicated a detection limit of 0.562 TCID_50_ with the EZ-SVV qRT-PCR (Ct value < 36). Therefore, a PCR result of < 36 was considered positive. Primers were designed based on the sequence of VP1 gene of SVV.

### ISH-RNA procedures

The ISH-RNA was performed using RNAscope, a RNA *in situ* hybridization technique [11]. Based on previously published qRT-PCR primers [3], ISH-RNA probe was developed by targeting a specific reverse complementary nucleotide sequence of the SVV (301–345 region of VP1 gene, GenBank: EU271758.1). Therefore, positive hybridization signals detected by ISH-RNA represent SVV mRNA that encodes VP1 protein. Although tissues infected with one of the four major swine vesicular diseases (SVD, VS, VES and FMD) were not available for evaluation of potential cross-reactivity by ISH-RNA, the ISH-RNA probe was designed from the same SVV genomic region of the previously described qRT-PCR assay (VP1 gene), which did not demonstrate cross-reaction against SVD, VS, VES and FMD [3]. Unspecific reaction was tested in positive samples for porcine epidemic diarrhea virus, transmissible gastroenteritis virus, rotaviruses (A, B and C), porcine circovirus type 2 and porcine reproductive respiratory syndrome virus, that are pathogens commonly found in pig farms. In general, individual tissues were tested by ISH-RNA. However, the individual qRT-PCR results were lacking for some tissues since the submitter pooled the tissues (lung, heart, spleen and kidney) (Table 1).

**Table 1:** Results of qRT-PCR and ISH-RNA in tissues from infected pigs. Ct values <36 were considered positive (+) and Ct values ≥36 were considered negative (-). *Ct values correspond to the pool of tissue homogenates (lung, heart, spleen and kidney). Specific and individual formalin-fixed tissues were used for ISH-RNA.

Pig ID		Snout vesicle		Brain		Heart		Lymph node		Kidney		Spleen		Lung		Liver		Colon		Small intestine		Tongue		Nasal sinus		Tonsil		Total
		ISH	qRT-PCR	ISH	qRT-PCR	ISH	qRT-PCR	ISH	qRT-PCR	ISH	qRT-PCR	ISH	qRT-PCR	ISH	qRT-PCR	ISH	qRT-PCR	ISH	qRT-PCR	ISH	qRT-PCR	ISH	qRT-PCR	ISH	qRT-PCR	ISH	qRT-PCR	
1	sow	+	13																									1
2	sow	+	12																									1
3	sow	+	13																									1
4	sow	+	12																									1
5	sow	+	12																									1
6	sow	+	16			-	33	+	23					-	30	-	29			-	28							6
7	sow			-	26	-	28	-	19	-	26			-	27													5
8	sow			-	26	+	28	+	28					+	26	-	24	+	33									6
9	piglet											+	30	+	30	+	31											3
10	piglet			-	33	-	24					+	21			+	21			-	25							5
11	piglet			-	30							+	17							+	24							3
12	piglet			-	34											+	25			-	28							3
13	piglet			-	33	+	29							+	27			+	30	+	30							5
14	piglet			-	32	-	27							+	27	+	26											4
15	piglet			-	32											-	32											2
16*	piglet													+	21	-	21					+	21	-	21			4
17*	piglet					-	25							-	25	-	25					+	25	-	25			5
18*	piglet																					+	23	-	23	+	23	3
Total		6		8		7		3		1		3		8		9		2		5		3		3		1		59

Unstained paraffin sections of tissue were deparaffinized in xylene and rehydrated through a series of alcohol washes. The rehydrated sections were tested by RNAscope technique (Advanced Cell Diagnostics Inc.) as previously described [11]. Briefly, tissues were treated with hydrogen peroxide at room temperature for 10 minutes. Then, sections were boiled (95° to 100°C) in citric buffer for 15 minutes and incubated with protease at 40°C for 30 minutes. The slides were hybridized with the SVV probe in hybridization buffer [6 SSC (1 SSC is 0.15 mol/L NaCl, 0.015 mol/L Na-citrate), 25% formamide, 0.2% lithium dodecyl sulfate, blocking reagents] at 40°C for 2 hours. Then, the sequence amplifiers were added for 15 or 30 minutes at 40°C. The red colorimetric staining detected the SVV hybridization signal and counterstaining occurred with hematoxylin. The microscopic assessment of a positive and negative result was conducted by two independent blinded pathologists.

## Results

The study selection resulted in 59 samples from 18 affected pigs and 19 samples from 15 non-affected pigs (total tissue, n = 78). A diverse set of organs were tested by ISH-RNA and 30 SVV qRT-PCR positive samples were also positive in ISH-RNA [snout vesicle (n = 6/6), heart (n = 2/7), lymph node (n = 2/3), spleen (n = 3/3), lung (n = 5/8), liver (n = 4/9), colon (n = 2/2), small intestine (n = 2/5), tongue (n = 3/3), tonsil (n = 1/1)] as enumerated in Table 1. With exception of one sample, all SVV qRT-PCR positive tissues with Ct values lower than 20 were positive for ISH-RNA. Samples with Ct values higher than 33 were negative by ISH-RNA. Hybridization signals were not present in the 19 samples from the non-affected pigs or in samples positive for porcine epidemic diarrhea virus, transmissible gastroenteritis virus, rotaviruses (A, B and C), porcine circovirus type 2 and porcine reproductive respiratory syndrome virus. These findings confirmed the specificity of the SVV probe for the targeted sequence. ISH-RNA results from SVV qRT-PCR positive samples according to the tissue-type and origin of samples (sows or piglets) are demonstrated in Table 1.

**Fig 1:**
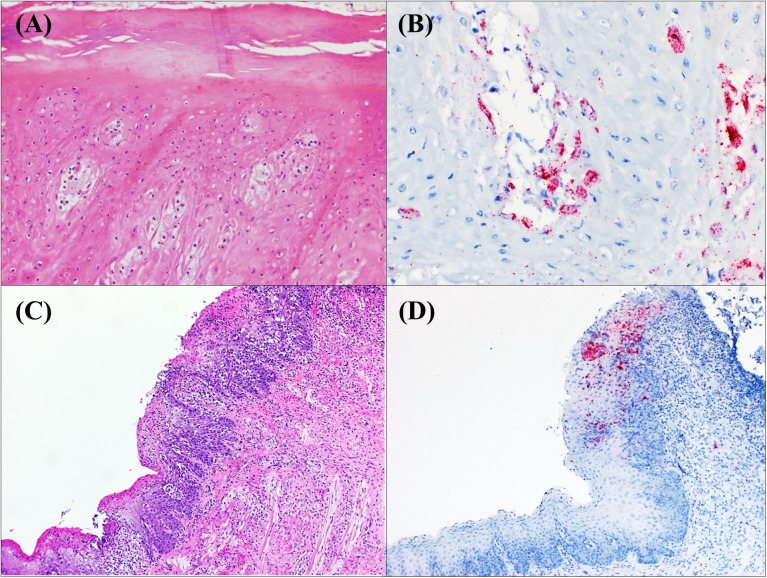
SVV distribution in vesicular lesions in the snout of an affected sow and in necrotizing lesions in tongue of an affected piglet. a) Skin from infected sow. Ballooning degeneration (intracellular edema) of keratinocytes in the *stratum spinosum* with formation of intraepidermal vesicles. H&E, 20x; b) Skin from infected sow. Intraepidermal vesicle showing strong SVV positive staining within the cytoplasm of keratinocytes. ISH-RNA, 40x; c) Tongue from infected piglet. Necrotizing glossitis. H&E, 20x; d) Tongue from the infected piglet shown in (c). Red dots and clusters represent the presence of SVV mRNA within an erosive lesion. ISH-RNA, 40x.

All the cutaneous tissues from the vesicular lesions (n = 6) on the snout of SVV affected sows were positive by ISH-RNA. SVV mRNA was more frequently observed in the *stratum spinosum* of the epidermis (Fig 1A and 1B). Interestingly, erosive lesions of tongues from piglets (pig ID 16, 17 and 18) were the only tissue from piglets that were positive for SVV staining in association with histological lesions (Fig 1C and 1D). Tissue fragments from the nasal sinuses from these piglets (pig ID 16, 17, 18) were negative by the ISH-RNA technique. A single tonsil was positive from these neonatal piglets (pig ID 18).

**Fig 2:**
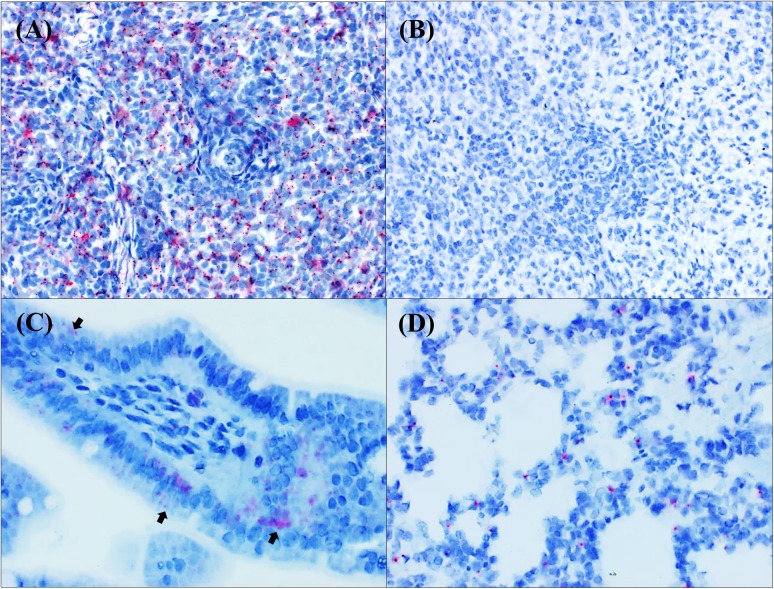
SVV distribution in tissues without evidence of histological lesions. Swine, ISH-RNA. a) Piglet, spleen (central arteriole). Strong SVV positive staining diffusely distributed throughout the splenic parenchyma. ISH-RNA, 20x.; b) Piglet, spleen. Negative control. ISH-RNA, 20x; c) Piglet, small intestine. SVV mRNA was multifocally distributed within enterocytes (black arrows) and lamina propria. ISH-RNA, 20x; d) Piglet, lung, SVV positive signals were found in alveolar septum. ISH-RNA, 20x.

Splenic samples with qRT-PCR positive results from affected piglets (pig ID 9, 10, 11) were ISH-RNA positive either clustered in small foci or diffusely distributed throughout the tissue section but they did not show any evidence of histological lesions (Fig 2). Lymph nodes from two sows with vesicular disease were positive by ISH-RNA and qRT-PCR with Ct 23 and Ct 28, respectively. Intriguing, another lymph node was strongly positive by qRT-PCR (Ct 19), but negative by ISH-RNA.

Myocardial samples had low number of positive ISH-RNA results (n = 2/7), but the distribution of SVV was characterized by small red dots diffusely distributed in the cytoplasm of cardiomyocytes. In the small and large intestines, virus was detected mainly in enterocytes’ cytoplasm (Fig 2C). In the lung, SVV infected cells were detected in alveolar septum of a single sow and four piglets (n = 5/9) (Fig 2D). The brain tissues from two sows and six piglets tested negative for SVV.

## Discussion

The novel RNA based *in situ* hybridization technique identified replication of SVV in tissue fragments of the tongue, tonsil, lung, heart, spleen, liver, small intestine, and colon, which has not been previously reported. In addition, this novel ISH-RNA platform is helpful in the diagnosis of emerging pathogens in the absence of a commercial antibodies for IHC. This ISH-RNA technique has been applied in research and diagnostics in human medicine [13,14] while in veterinary medicine, ISH-RNA platform was used in tissues from cattle, pigs, and dogs [15–19] in the context of various infectious diseases.

Clinical signs of SVV are indistinguishable from other important vesicular diseases, such as foot-and-mouth disease, which is considered by the World Organization for Animal Health (OIE) as one of the most contagious disease of animal, with the potential to result in devastating economic losses. Consequently, SVV outbreaks would have significant impacts on the US pork industry, such as temporary closures of pork processing plants, resource re-allocation for disease investigations, increased pre-weaning mortality and increased sow culling rates. Based on the recent US outbreak, the SVV ISH-RNA played a critical role as a rapid diagnostic response by supporting the SVV qRT-PCR.

The ISH-RNA technique for SVV established the association between histological lesions and the presence of the virus, with minimal background and non-specific staining.

Three lymph node samples (pig ID 6, 7, and 8) were analyzed by ISH-RNA. The samples with relatively high Ct value (Ct = 28, pig ID 8) was ISH-RNA positive, while the sample with low Ct values (Ct = 19, pig ID 7) was ISH-RNA negative. We speculate that qRT-PCR detected virus being processed by professional antigen-presenting cells, but not replicating actively virus particles. Conversely, the two lymph nodes showing positive staining detected by ISH-RNA would represent actively replicating SVV characterized by expression of virus mRNA.

As expected, SVV ISH-RNA positive cells were observed in all snout vesicles (tissues with lowest Ct values). Interestingly, positive SVV cells were found in tissues of tongue from piglets showing erosive glossitis while negative SVV results were obtained in tissue sections of tongue without lesions, similarly to other reports [20] that used IHC to screen SVV antigens in pig tissues. Erosive glossitis is commonly found in pre-weaning piglets as a consequence of mechanical trauma from environmental surfaces. Therefore, SVV may also be present in lesions of tongue as an opportunistic infection after SVV viremia. Recent reports described the gross and microscopic lesions in different tissues in ETNL-affected piglets in Brazil [5,20]. However, absence of lesions in ETNL cases in Brazil and USA have also been reported [3,6,21].

The lack of histological lesions associated with high levels of SVV in tissues from affected piglets was a critical feature, which needs further clarification. The pathogenesis associated with mortality in neonatal piglets has not been reported to date. Vesicular disease was recently demonstrated in 9- and 15-week old pigs experimentally infected with SVV [18,22]. A recent case-control study reported neonatal mortality associated with SVV infection without vesicular disease in sows, in 43% of the affected herds [21]. Despite these recent studies, ETNL has not been experimentally reproduced. Therefore, the hypothesis of SVV being a primary causative agent for acute neonatal mortality remains to be elucidated.

The present study detected SVV using ISH in various tissues in affected piglets. Our findings corroborate with previous study of high SVV titer detected by qRT-PCR [3,6]. Diarrhea and mesocolonic edema have been frequently reported in ETNL-affected piglets [20,21]. Our results identified SVV mRNA within the cytoplasm of enterocytes and colonocytes, and in the lamina propria of the intestine. However, there was no evidence of histological lesions in these tissues. This study did not intend to investigate the pathogenesis of SVV in neonatal piglets, but the ISH-RNA presented here illustrated the replication of SVV in affected piglets, including cardiomyocytes. Myotropism in FMD virus has been well studied in several species [23]. In addition, myocardial necrosis and myocarditis were previously documented in FMD-affected pigs [24] and in SVV-affected piglets [20]. In contrast, our study did not find any lesions associated with the presence of SVV in the myocardium.

Furthermore, although we have observed SVV positive signals in lungs (5/8), no histological lesions were found. Opposite situation has been reported in piglets from different regions in Brazil [20]. Although pulmonary edema, congestion and interstitial pneumonia were diagnosed, no SVV antigen was detected by IHC in affected samples [20]. Tonsils have been proved by ISH-RNA to be an important site of SVV replication in experimentally infected pigs [18]. Still, due to the diversity of tissues in our study, we have found only one ISH-RNA positive tonsil (n = 1/1) from a piglet (pig ID 18), but we did not test any tonsil from affected sows.

False positive staining was considered an important limitation of classical *in situ* hybridization. The ISH-RNA technique developed for SVV detection greatly removed this constraint, based on the lack of non-specific staining in sections negative by PCR, and there was no difficulty in interpretation of specific SVV staining in positive samples.

SVV-ISH-RNA showed high specificity, but the sensitivity was not equivalent to the qRT-PCR assay. Nevertheless, while qRT-PCR detects genomic RNA from viable or non-viable virus particles in lysed homogenate of fresh tissues, ISH allows the identification of metabolically active virus through detection of viral mRNA within morphologically preserved tissues sections. The combination of qRT-PCR and ISH will result in a high sensitive and specific approach to diagnosis SVV in pig samples with a morphological context.

## Conclusions

In conclusion, an ISH-RNA technique for SVV was established in this study and demonstrated high level of specificity. This novel *in situ* hybridization platform allows the association of SVV and histological lesions, which complements qRT-PCR as a diagnostic technique to investigate potential emerging pathogens. The ISH-RNA offers a simple and effective early diagnostic technique for SVV in sows with vesicular disease and in acute neonatal mortality cases. The ISH-RNA technique is also a potential methodology to improve the knowledge on the pathogenesis of SVV, especially in ETNL-affected animals.

## Supporting information

S1 Table qRT-PCR negative samples used to verify non-specific staining. (DOCX)
