# Supplementary material for: A novel RNA-based in situ hybridization to detect Seneca Valley virus in neonatal piglets and sows affected with vesicular disease
Source: PLoS One. 2017 Apr 10;12(4):e0173190. doi: 10.1371/journal.pone.0173190 (PMC5386259; doi:10.1371/journal.pone.0173190)
Supplement: S1 Table — (DOCX) [file pone.0173190.s001.docx]

| **Animal ID** | **Category** | **skin** | | **tongue** | | **liver** | | **lymph node** | | **spleen** | | **lung** | |
| --- | --- | --- | --- | --- | --- | --- | --- | --- | --- | --- | --- | --- | --- |
|  |  | **ISH** | **PCR** | **ISH** | **PCR** | **ISH** | **PCR** | **ISH** | **PCR** | **ISH** | **PCR** | **ISH** | **PCR** |
| **19** | **sow** | - | - |  |  |  |  |  |  |  |  |  |  |
| **20** | **sow** | - | - |  |  |  |  |  |  |  |  |  |  |
| **21** | **sow** | - | - |  |  |  |  |  |  |  |  |  |  |
| **22** | **sow** | - | - |  |  |  |  |  |  |  |  |  |  |
| **23** | **sow** | - | - |  |  |  |  |  |  |  |  |  |  |
| **24** | **sow** | - | - |  |  |  |  |  |  |  |  |  |  |
| **25** | **sow** | - | - |  |  |  |  |  |  |  |  |  |  |
| **26** | **sow** |  |  |  |  | - | - | - | - | - | - | - | - |
| **27** | **piglet** |  |  | - | - |  |  |  |  |  |  |  |  |
| **28** | **piglet** |  |  | - | - |  |  |  |  |  |  |  |  |
| **29** | **piglet** |  |  | - | - |  |  |  |  |  |  |  |  |
| **30** | **piglet** |  |  |  |  | - | - |  |  | - | - |  |  |
| **31** | **piglet** |  |  |  |  |  |  |  |  | - | - |  |  |
| **32** | **piglet** |  |  |  |  |  |  | - | - |  |  |  |  |
| **33** | **piglet** |  |  |  |  |  |  | - | - |  |  |  |  |

**Supplementary File -** **PCR negative samples used to verify non-specific staining**
